# Workforce and Contents of Home Dental Care in Japanese Insurance System

**DOI:** 10.1155/2020/7316796

**Published:** 2020-07-26

**Authors:** Yoshiaki Nomura, Ayako Okada, Erika Kakuta, Ryoko Otsuka, Hideaki Saito, Hiroshi Maekawa, Hideki Daikoku, Nobuhiro Hanada, Tamotsu Sato

**Affiliations:** ^1^Department of Translational Research, Tsurumi University School of Dental Medicine, 2-1-3 Tsurumi Tsurumi-ku, Yokohama 230-8501, Japan; ^2^Department of Oral Microbiology, Tsurumi University School of Dental Medicine, 2-1-3 Tsurumi Tsurumi-ku, Yokohama 230-8501, Japan; ^3^Department of Operative Dentistry, Tsurumi University School of Dental Medicine, 2-1-3 Tsurumi Tsurumi-ku, Yokohama 230-8501, Japan; ^4^Iwate Dental Association, 2-5-25 Morioka-eki-Nishi-dori, Morioka 020-0045, Japan

## Abstract

**Background:**

In Japan's super-aging society, it is required to establish a home dental service provision system. It is necessary to analyze the current state of visiting dentistry.

**Methods:**

A cross-sectional survey was conducted using a self-administered postal mail questionnaire distributed to all members of the Iwate Dental Association. We analyzed the implementation status of dental care at home/nursing facilities, the number of dental clinic staff, and the contents of dental home care. Correspondence analysis, item response theory, and zero-inflated model were used for the analysis.

**Results:**

Of the 354 dental clinics, 187 (52.8%) performed visiting dental care and 195 (55.1%) implemented dental care in a nursing home. Visit dentistry was mainly performed by part-time workers. Denture treatment and oral care were common treatments for dental home care.

**Conclusion:**

More than half of the dental clinics did not offer visiting dentistry. In order to fully provide dental visiting services, infrastructure development is necessary. Specifically, human resources are most important, even if they are part-time workers.

## 1. Introduction

Japan is currently a super-aged society. In February 2019, the population over the age of 65 was 35.5 million, accounting for 28.2% of the total population [1]. The life expectancy of a 65-year-old man was 19.57 years and that of a woman was 24.43 years [2]. According to a national survey conducted every five years, the number of remaining teeth in the age group that are from 65 to 69 years old was 21.9. The number of remaining teeth was 10.7 in the age group of 85 years or older. The number of remaining teeth in the elderly is increasing [3]. For these people, dental care and oral care are essential to promoting their healthy life and quality of life. Therefore, the demand for visiting home dental care will increase.

In 2016, the number of Japanese dentists was 104,533 [4]. Of these, 89,166 (85.3%) worked in dental clinics. The number of dental clinics in Japan was 69,592 in 2014 [5]. According to these statistics, one dentist needs to care for 400 old people, and one dental clinic needs to care for 512 old people. The average number of dentists at one dental office was 1.28. The majority of the clinics have only one dentist.

The estimated number of outpatients receiving home health care was 156.4 thousand per day. 14.4 thousand patients received home care from hospital visits, 101.5 thousand patients received home care from clinic visits, and 40.6 thousand patients received home care from dental clinic visits. Of the 40.6 thousand patients, 31.5 thousand were 65 years or older [6]. Only 0.09% of seniors aged 65 and over used home dental services in one day. This fact may indicate that the home dentistry service supply system is not sufficient.

While primary care dentists contributed to the subjective well-being of the elderly [7], they experienced barriers in several areas: lack of knowledge [8], inadequate training and experience [9, 10], actual situation [8], lack of proper equipment [9, 10], loss of time [11].

Therefore, it is necessary to analyze the current situation of home dental care. In this study, we analyzed the relationship between the scale of dental clinics and the implementation of dental care in home and nursing care facilities. We also analyzed the contents of home dental treatment.

## 2. Methods

### 2.1. Study Population

This survey was a cross-sectional survey conducted using a self-filled mail questionnaire. The questionnaire was distributed to all 609 members of the Iwate Dental Association.

### 2.2. Questionnaire

The survey consisted of 61 main categories. The 61 major categories were mainly demographic factors, the size of the dental clinic, the implementation of visiting dentistry, the medical and dental collaboration, and the availability of services provided by the Iwate Dental Association to its members.

Of these items, the following were used in the analysis: the implementation of home dentistry, the frequency of visits and the content of home dentistry, the place to visit home dentistry (home or nursing home), and the clinic's number of dental staff.

### 2.3. Data Analysis

A cross-tabulation was performed on the location and frequency of visit dental services. Correspondence analysis was performed by this cross-tabulation. The results were illustrated graphically as a biplot [11]. A three-parameter logistic model was applied under the Item Response Theory (IRT) approach [12–15]. Item difficulty, item discrimination, guessing, and item information were calculated. The item response curve and the item information curve were illustrated.

The average number of dental staff was calculated against the implementation of visiting dental care or not. We used a zero inflation negative binomial model consisting of two components. The first component was governed by a binary distribution that produced structural zeros. The second component was a negative binomial distribution that produced counts [16, 17].

Analysis of IRT was performed by the *R* software with ltm and irtoys library. Analysis of zero-inflated model was performed by Glm, AER, glm2, pscl, and MASS library. Other analyses including descriptive statistics and correspondence analysis were conducted with IBM SPSS Statistics version 24 (IBM Inc., Tokyo, Japan).

### 2.4. Ethics Statement

The survey was conducted in accordance with the principles of the Declaration of Helsinki. When the questionnaire was returned, each subject obtained their written consent for publication. This study was approved by the Ethics Committee of the Faculty of Dentistry, Tsurumi University (approval number: 1730).

## 3. Results

The questionnaire was distributed to all 609 members of the Iwate Dental Association and was collected from 354 (collection rate 58.1%). Dental clinics in 187/354 (52.8%) performed visiting home dental care, and 195/354 (55.1%) performed dental care in a nursing home.

Table 1 shows the annual number of dental procedures performed visiting dental care at home, nursing homes, and both home and nursing home dental clinics. Denture treatment was the most common (201/354 : 57.1%), followed by oral care (119/354 : 33.8%). Dental caries (87/354 : 24.7%), periodontal disease (83/354 : 23.1%), and oral surgery including tooth extraction (71/354 : 20.1%) were less than 25%.

The relationship between each treatment and implemented place was graphically presented as a biplot analyzed by correspondence analysis. Denture treatment was located between home and nursing home. Oral care was centrally located between home, nursing home, and both home and nursing home. Both caries and oral surgery were located away from home.

**Figure 1:**
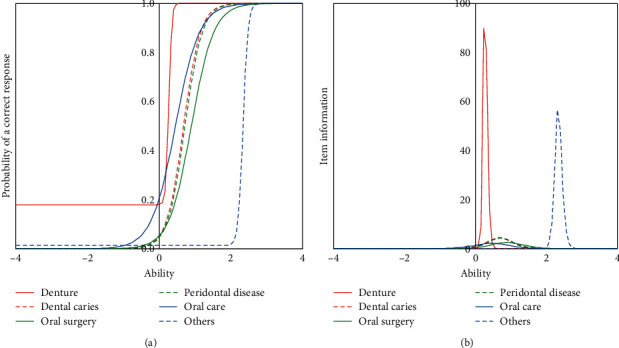
Item response curves and item information curves of the home dental care treatment.

The characteristics of these treatments were analyzed by a three-parameter logistic model of the item response theory (IRT) approach. The model is shown in Table 2. The item response curve and item information curve are shown in Figure 1. The guessing for denture treatment was 0.18, continuing across the *Y* axis. It had very high item information. The caries treatment and periodontal treatment showed similar patterns. The item response curve of oral care shifted to the left from these two items. Oral surgery, including tooth extraction, shifted to the right.

The characteristics of individual treatments were analyzed by a three-parameter logistic model of the item response theory (IRT) approach. The model is shown in Table 2. The item response curve and item information curve are shown in Figure 1. The estimate for denture treatment is 0.18, which is across the *Y* axis. There was a great deal of item information on denture treatment. Caries treatment and periodontal treatment showed similar patterns. The item response curve of oral care was shifted to the left of these two items. Oral surgery including tooth extraction shifted to the right.

**Figure 2:**
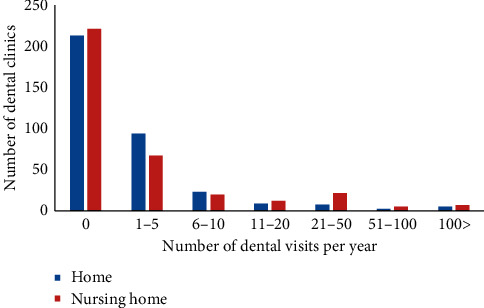
Histogram of the number of dental visits per year. Both at home and nursing home showed skewed distribution.

The size of the dental office, including human resources, can impact the practice of visiting home dentistry. As a descriptive analysis, the number of dental staff was compared by the presence or absence of home dental care. As shown in Table 3, the number of dental hygienists and dental assistants had a major impact on the practice of home dentistry.  Figure 2 shows a histogram of the number of home dental care deliveries per year. The number of implementations varied greatly from 0 to 187 at home and from 0 to 1951 at nursing homes. A zero inflation model was applied for statistical modeling. The results are shown in Table 4. The zero-inflated negative binomial model uses two components. The first component is governed by a binary distribution that produces zero. The second component is the negative binomial distribution that produces the count. In the case of dental home care, implementation was heavily dependent on the number of part-time dental hygienists and visits were independent of the number of dental staff. In contrast, when visiting a nursing home, implants had a negative relationship with the number of regular workers in dental hygienists and assistants. And it was positively associated with the number of part-time dental assistants. The number of implementations was negatively associated with full-time workers of dental hygienists and dental assistants and positively associated with part-time workers of dental hygienists and dental assistants.

## 4. Discussion

In this study, we analyzed the contents of home dental care and the relation between the home dental care and the number of dental staff.

Table 1 shows descriptive statistics on the frequency of home dental care. The frequency of visiting was higher in dental clinics that provided dental care both at home and in nursing homes than in homes or nursing homes alone. Dental clinics that visit both homes and nursing homes may be able to handle a variety of dental treatments.

**Figure 3:**
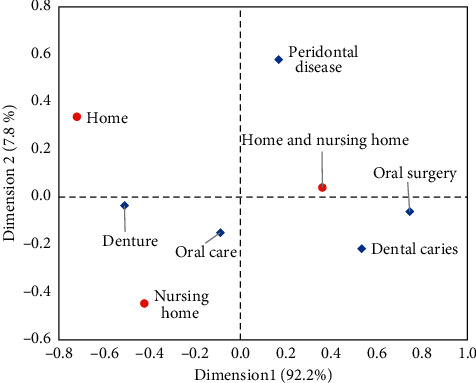
Biplot by correspondence analysis of implemented place and contents of visiting dental treatment.

Firstly, we focused on the contents of home dental care and the difficulty for the implementation of each content. As shown in Figure 3, dental caries and oral surgery are located far from the dental clinic, which only visits the home. Treatment of caries may require mobile-specific devices [9, 10]. In some cases, air turbines are essential for removing caries. Oral surgery treatment requires knowledge and experience in general health monitoring. Knowledge of medical conditions is also required. Previous studies have shown that knowledge and experience were one of the obstacles to home dental care [8]. These facts suggest that there may be some barriers and limitations to a small dental clinic for home dental care.

Denture treatment was located between a dental clinic that provides home dental treatment and a nursing home. The item response curve for denture treatment showed that even dental clinics, which rarely do home dentistry, can handle denture treatment. The slope of the item response curve for denture treatment was steep, and the item information was very high. Most dental clinics that can handle caries, periodontal disease, and oral surgery may be able to handle denture treatment.

Therefore, denture treatment was the most common treatment for home dentistry. The difficulty level of oral care shown in Table 2 was lower than that of caries, periodontal disease, and oral surgery. Oral care was the second most common dental treatment. This fact indicates that some older people received only denture treatment without oral care.

Next, we analyzed the dental staffs that provide the home dental care. As shown in Figure 2, more than half of the dental clinics did not provide home dental care. The percentage was 60% for home care and 63% for nursing home care. Most frequent visits were less than 5 times a year, with 26% in home dental care and 19% in nursing home care. This fact indicates that most dental clinics provide home dental care to one or two patients each year. The Iwate Dental Association assigns visiting dental treatments to members at the request of nursing homes and local governments. Clinics belonging to the Iwate Dental Association must perform home dental care, even if it is not their primary concern. Comparing home dental care with nursing home dental care, the time loss of home dental care is very large. Nursing homes can treat several patients in a single visit. Therefore, as shown in Table 3, the implementation of home dental treatment did not depend on the number of dental staffs. For dental care in nursing homes, the dental clinic requires a contract with a nursing home, and the dental clinic is obliged to visit the nursing home regularly. Therefore, dental care in nursing homes requires an infrastructure that includes dental staff.

As a result of the zero-inflated model analysis, the practice of home dentistry relied on dental hygienists and dental assistants. The number of full-time employees of dental hygienists and dental assistants was negatively associated with home dentistry practices. Full-time workers can provide home-visit dentistry. This result suggests that, in many dental clinics, home dental care is performed by part-time workers and not regular workers.

In conclusion, more than half of the dental clinics surveyed did not provide home dental care. In order to fully provide home dental care services, it is necessary to improve the infrastructure, including increasing the number of staff.

Denture treatment was located between at home and nursing home. Oral care was centrally located at three implementation sites. Treatment of dental caries, periodontal disease, and oral surgery was centrally located both at home and nursing home.

Guessing of denture treatment was 0.18 and it continued across the *Y* axis. Denture treatment had very high item information. Dental caries treatment and periodontal disease treatment showed similar pattern. Item response curve of oral care shifted left side from these two items. Oral surgery including tooth extraction shifted for right side.

## Figures and Tables

**Table 1:** Frequency of place and contents of home dental care.

		Home	Nursing home	Home and nursing home	Total	*P* value
Denture	−	10	5	4	152	<0.001
	+	52	49	100	201	

Dental caries	−	53	39	41	266	<0.001
	+	9	15	63	87	

Oral surgery	−	56	44	49	282	<0.001
	+	6	10	55	71	

Periodontal disease	−	45	43	49	270	<0.001
	+	17	11	55	83	

Oral care	−	39	28	34	234	<0.001
	+	23	26	70	119	

Others	−	59	54	98	344	0.014
	+	3	0	6	9	

*P* values were calculated by *χ*^2^ tests.

**Table 2:** Results of three-parameter logistic model for the contents of home dental care.

	Discrimination	Difficulty	Guessing
Denture	25.47	0.26	0.18
Dental caries	4.26	0.68	<0.001
Oral surgery	3.22	0.90	<0.001
Periodontal disease	4.18	0.72	<0.001
Oral care	2.99	0.45	<0.001
Others	15.47	2.34	0.01

Parameters were estimated by maximum likelihood estimation using the following formula:*P*_*i*_(*θ*)=*C*_*i*_+(1 − *c*_*i*_)/1+*e*_*i*_(*θ* − *b*_*i*_), *a*_*i*_: discrimination parameter, *b*_*i*_: difficulty parameter, *c*_*i*_: guessing parameter.

**Table 3:** Number of dental staffs with or without implementation of home dental care.

		Home dental care					
		Home			Nursing home		
		−	**+**	*P* value	−	**+**	*P* value
Dentist	Regular	1.20+/−0.49	1.35+/−0.71	0.024	1.27+/−0.57	1.28+/−0.66	0.948
	Part-time	0.30+/−0.74	0.37+/−0.86	0.424	0.28+/−0.74	0.40+/−0.87	0.078

Dental hygienist	Regular	1.16+/−1.12	1.52+/−1.53	0.030	1.19+/−1.30	1.50+/−1.38	0.017
	Part-time	0.36+/−0.74	0.44+/−0.77	0.191	0.34+/−0.73	0.46+/−0.78	0.082

Dental assistant	Regular	1.27+/−1.22	1.59+/−1.29	0.009	1.29+/−1.28	1.58+/−1.24	0.007
	Part-time	0.33+/−0.72	0.37+/−0.79	0.927	0.33+/−0.77	0.37+/−0.73	0.204

*P* values were calculated by Mann–Whitney *U* tests. Numbers of regular attendees of dental hygienists and dental assistants were statistically significant.

**Table 4:** Zero-inflated model for number of home dental care visits predicted by human resources.

		Dental care					
		Home			Nursing home		
		Estimate	SD	*P* value	Estimate	SD	*P* value
Count model (negative binominal, log link): number of visits per year							
	(Intercept)	0.97	0.42	0.021	3.30	0.48	<0.001
Dentist	Regular	0.39	0.27	0.146	0.05	0.26	0.864
	Part-time	−0.07	0.31	0.811	0.04	0.23	0.858
Dental hygienist	Regular	0.003	0.14	0.981	−0.53	0.15	<0.001
	Part-time	0.63	0.27	0.019	0.74	0.22	0.001
Dental assistant	Regular	−0.10	0.16	0.527	−0.54	0.17	0.001
	Part-time	−0.05	0.25	0.857	1.44	0.34	<0.001

Zero-inflation model: implementation or not							
	(Intercept)	−79.94	66121	0.999	0.98	1.00	0.325
Dentist	Regular	81.22	66121	0.999	0.42	0.86	0.625
	Part-time	52.45	33061	0.999	0.05	0.32	0.864
Dental hygienist	Regular	−47.58	33061	0.999	−1.33	0.44	0.003
	Part-time	−0.36	0.68	0.599	−0.12	0.28	0.668
Dental assistant	Regular	−84.12	66121	0.999	−1.16	0.49	0.018
	Part-time	−59.05	33061	0.999	0.75	0.37	0.041
AIC	1344.597		1467.303				

## Data Availability

All relevant data are administrated by Iwate Dental Association. Data can be provided upon reasonable request.

## Supplementary Materials

Supplementary Materials S Table: the questionnaire used in this study.
