# Supplementary material for: Workforce and Contents of Home Dental Care in Japanese Insurance System
Source: Int J Dent. 2020 Jul 26;2020:7316796. doi: 10.1155/2020/7316796 (PMC7399789; doi:10.1155/2020/7316796)
Supplement: Supplementary Materials — S Table: the questionnaire used in this study. [file 7316796.f1.docx]

S Table The questionnaire used in this study

Check the number of staffs of your clinic

|  | Regular worker | Part time |
| --- | --- | --- |
| Dentist | □1，□2，□3，□4，□5，□（　　） | □0，□1，□2，□3，□4，□5，□（　　） |
| Dental hygienist | □0，□1，□2，□3，□4，□5，□（　　） | □0，□1，□2，□3，□4，□5，□（　　） |
| Dental technician | □0，□1，□2，□3，□4，□5，□（　　） | □0，□1，□2，□3，□4，□5，□（　　） |
| Dental assistant | □0，□1，□2，□3，□4，□5，□（　　） | □0，□1，□2，□3，□4，□5，□（　　） |
| Others | □0，□1，□2，□3，□4，□5，□（　　） | □0，□1，□2，□3，□4，□5，□（　　） |

Answer the questions about implementation of visiting dental care last year

| Implementation of visiting dental care last year | |
| --- | --- |
| Implemented at patients’ home | □ Yes, □ No |
| Implemented at nursing home | □ Yes, □ No |
| Fill the number of implementation per year | |
| At patients’ home | [ ] |
| At nursing home | [ ] |
| Check the contents of visiting dental treatment | |
| Denture | □ Yes, □ No |
| Dental caries treatment | □ Yes, □ No |
| Oral surgery | □ Yes, □ No |
| Periodontal treatment | □ Yes, □ No |
| Oral care | □ Yes, □ No |
| Others | □ Yes, □ No |
